# L-type amino acid transporter 1 is associated with chemoresistance in breast cancer via the promotion of amino acid metabolism

**DOI:** 10.1038/s41598-020-80668-5

**Published:** 2021-01-12

**Authors:** Miku Sato, Narumi Harada-Shoji, Takafumi Toyohara, Tomoyoshi Soga, Masatoshi Itoh, Minoru Miyashita, Hiroshi Tada, Masakazu Amari, Naohiko Anzai, Shozo Furumoto, Takaaki Abe, Takashi Suzuki, Takanori Ishida, Hironobu Sasano

**Affiliations:** 1 Department of Breast and Endocrine Surgical Oncology, Tohoku University Graduate School of Medicine, 1-1 Seiryo-machi, Aoba-ku, Sendai, 980-8574 Japan; 2 Department of Pathology, Tohoku University Hospital, 1-1 Seiryo-machi, Aoba-ku, Sendai, 980-8574 Japan; 3 Division of Nephrology, Endocrinology, and Vascular Medicine, Tohoku University Graduate School of Medicine, 1-1 Seiryo-machi, Aoba-ku, Sendai, 980-8574 Japan; 4 Institute for Advanced Biosciences, Keio University, Tsuruoka, Yamagata 997-0035 Japan; 5 Sendai Medical Imaging Center, 2-1-25 Itsutsubashi, Aoba-ku, Sendai, 980-0022 Japan; 6 Department of Breast Surgery, Tohoku Kosai Hospital, 2-3-11 Kokubuncho, Aoba-ku, Sendai, 980-0803 Japan; 7 Department of Pharmacology, Chiba University Graduate School of Medicine, 1-8-1 Inohana, Chiba, 260-0856 Japan; 8 Cyclotron and Radioisotope Center, Tohoku University School of Medicine, 6-3 Aramaki-aza-Aoba, Aoba-ku, Sendai, 980-8578 Japan; 9 Department of Pathology and Histotechonology, Tohoku University Graduate School of Medicine, Tohoku University Hospital, 1-1 Seiryo-machi, Aoba-ku, Sendai, 980-8574 Japan

**Keywords:** Cancer, Endocrinology, Medical research, Oncology

## Abstract

^18^F-FDG PET/CT has been used as an indicator of chemotherapy effects, but cancer cells can remain even when no FDG uptake is detected, indicating the importance of exploring other metabolomic pathways. Therefore, we explored the amino acid metabolism, including L-type amino acid transporter-1 (LAT1), in breast cancer tissues and clarified the role of LAT1 in therapeutic resistance and clinical outcomes of patients. We evaluated LAT1 expression before and after neoadjuvant chemotherapy and examined the correlation of glucose uptake using FDG-PET with the pathological response of patients. It revealed that LAT1 levels correlated with proliferation after chemotherapy, and amino acid and glucose metabolism were closely correlated. In addition, LAT1 was considered to be involved in treatment resistance and sensitivity only in luminal type breast cancer. Results of in vitro analyses revealed that LAT1 promoted amino acid uptake, which contributed to energy production by supplying amino acids to the TCA cycle. However, in MCF-7 cells treated with chemotherapeutic agents, oncometabolites and branched-chain amino acids also played a pivotal role in energy production and drug resistance, despite decreased glucose metabolism. In conclusion, LAT1 was involved in drug resistance and could be a novel therapeutic target against chemotherapy resistance in luminal type breast cancer.

## Introduction

Neoadjuvant chemotherapy (NAC) has become an important therapeutic option for breast cancer. At present, NAC is administered not only to patients with advanced-stage breast cancer but also to those harboring early or operable cancer because NAC improves the rate of breast conservation and decreases the extent of intraoperative axillary exploration^1^. In addition, patients with estrogen receptor (ER)-negative and/or human epidermal growth factor receptor-2 (HER2)-positive breast cancer who achieve pathological complete response (pCR) following NAC have better clinical outcomes^2,3^. Moreover, the clinical benefits of NAC have been reported in luminal type breast cancer; however, the therapeutic effects of chemotherapy are significantly lower among patients with the luminal subtype than among patients with other subtypes, and pCR is not necessarily correlated with clinical outcome^4^. Therefore, it is critical to explore surrogate markers that could predict the clinical outcome of patients with luminal type breast cancers before administering NAC.

Glycolysis in carcinoma cells is crucial because rapid cell proliferation requires biosynthetic energy^5,6^. [18F] Fluoro-2-deoxy-D-glucose (FDG)-PET assesses glucose uptake by detecting the uptake of the glucose analog, FDG, by glucose transporters^7–9^. The RECIST criteria have been widely used for evaluating the clinical response to chemotherapy in solid tumors^10^. However, FDG-PET/CT has been more frequently used in assessing the therapeutic effects of chemotherapy in breast cancer patients^11^ because the reduction in FDG uptake is more rapid than morphological changes^12,13^. Therefore, the Positron Emission Tomography Response Evaluation Criteria in Solid Tumors, which are based on FDG-PET/CT scanning, have become clinical markers of chemotherapy efficacy^14^. However, breast carcinoma cells persist in more than half of patients who display no FDG uptake or in those who achieve complete metabolic response (CMR)^15^, indicating that breast cancer cells utilize metabolomic pathways besides that of glucose.

To maintain cell growth in cancer cells, the diverse energy producing pathways is clearly beneficial for its growth and survival. Therefore, the capability to switch from glucose to another fuel source to supply TCA intermediates to use mitochondrial oxidative phosphorylation (OXPHOS) for ATP production provides a clear survival advantage^16^. TCA intermediates, such as fumarate, succinate, and 2-hydroxyglutarate, which is produced by mutant isocitrate dehydrogenase, are collectively referred to as oncometabolites and contribute to the development and progression of cancer^17^. In addition, the supply of amino acids to the TCA cycle has been reported to increase in various human malignancies^18,19^. In particular, essential amino acids (EAAs) that are imported by various amino acid transporters are important for proliferation because cancer cells rapidly die upon the removal of even a single EAA in vitro^20^. The L-type amino acid transporter (LAT) family is a pivotal uptake route of EAAs and consists of four members (LAT 1–4); LAT1 has been considered especially crucial to cancer biology and has a higher expression in cancer cells than in normal cells^19,21^. LAT1 transports many EAAs, such as leucine, isoleucine, valine, phenylalanine, tyrosine, tryptophan, methionine, and histidine^22^. LAT1 expression levels are correlated with adverse clinical outcomes in cancer patients, and a randomized controlled phase 2 clinical trial of LAT1 inhibitors (JPH203) is in progress in patients with advanced biliary tract cancers^23^.

ER-positive breast carcinoma cells have been reported to require leucine, which is transported by LAT1, increasingly, by binding to the scaffolding protein LLGL2, for proliferation^24^. The overexpression of both LLGL2 and LAT1 in carcinoma cells has been proposed as a mechanism of resistance to endocrine therapy^24^. However, little is known about the biological or clinical significance of these amino acid transporters with regard to the therapeutic effects of or resistance to chemotherapy in breast cancer patients. Therefore, in this study, we aimed to explore in situ amino acid metabolism, including LAT1, in breast cancer and to clarify the roles of LAT1 in the development of therapeutic resistance and clinical outcomes.

## Results

### Clinicopathologic characteristics and their association with LAT1 expression

The clinicopathological characters of the patients are summarized in Table 1. In this study, of 143 patients who received NAC, 123 patients (86.0%) had lymph node metastasis and 129 (90.2%) had received both anthracyclines and taxanes. Thirty-seven patients (25.9%) achieved pCR after NAC based on histopathological evaluation.

**Table 1:** Clinicopathological factors of breast cancer patients with chemotherapy before surgery (n = 143).

Factors	Number	Percentage (%)
Median age, (years)	56	
(Range)	(29–79)	
**cT status**		
1/2/3/4	24/86/8/25	16.8/60.1/5.6/17.5
**cN status**		
NO/1/2/3	20/105/6/12	14.0/73.4/4.2/8.4
**cStage status**		
I//II/III	8/96/39	5.6/67.1/27.3
**Pre subtype**		
ER positive	88	61.5
HER2 positive	37	25.9
Triple-negative	39	27.3
Pre Ki-67 LI	43.6	
Median (%)	(1.5–95)	
**Regimen of chemotherapy**		
Anthracycline and Taxane	103	72.0
Anthracycline , Taxane and Trastuzmab	26	18.2
Anthracycline only	12	8.4
Taxane only	2	1.4
**Pathological response**		
Grade la/lb/2a/2b/3	27/42/27/10/37	18.9/29.3/18.9/7.0/25.9

ER, estrogen receptor; HER2, human epidermal growth factor receptor-2.

**Figure 1:**
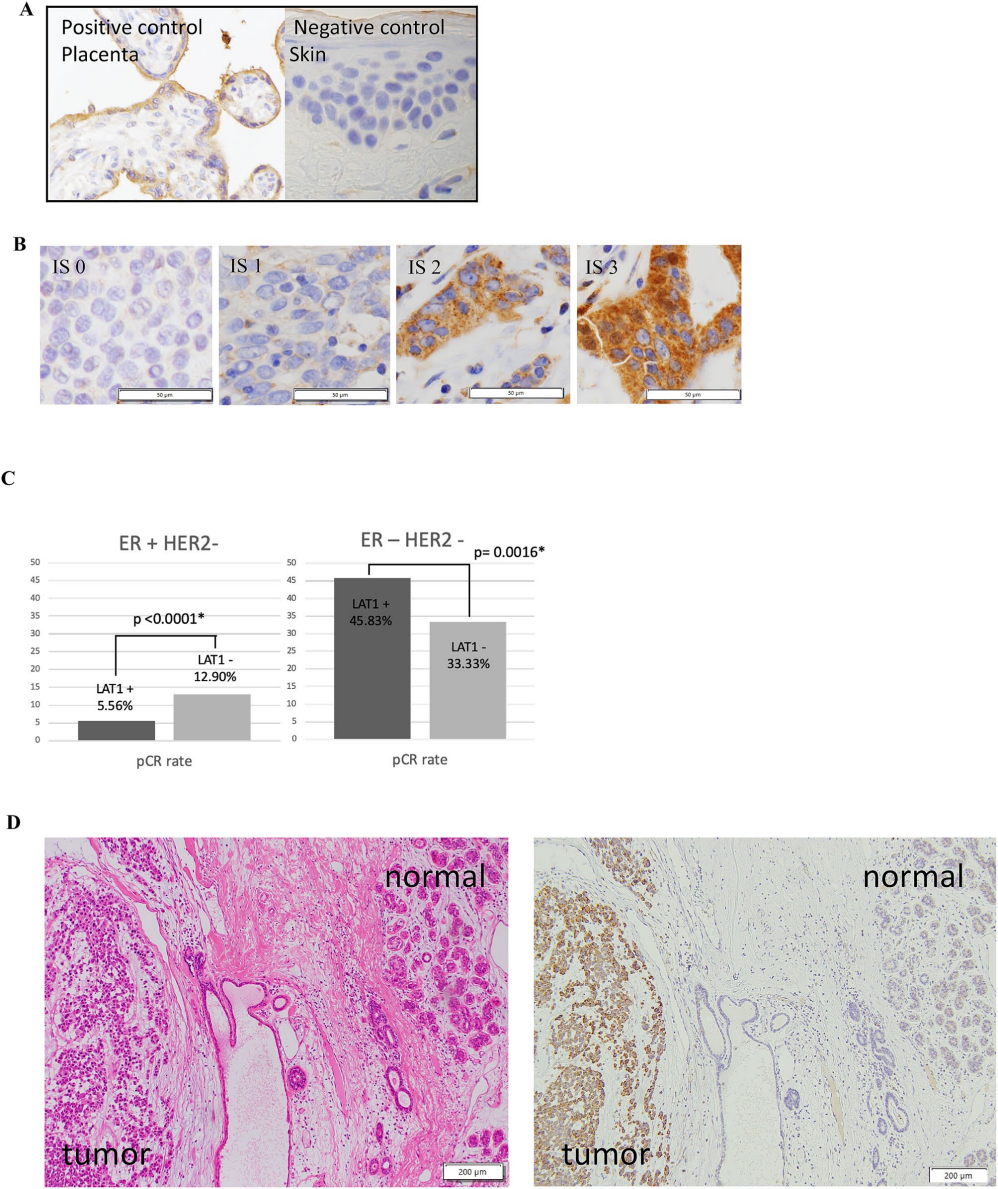
Immunohistochemistry for LAT1. Human placenta and skin tissue were used as positive and negative control (**A**). Immunohistochemical staining of LAT1. Intensity were scored from 0 to 3 (0: no membranous staining, 1: low intensity, 2: weak to moderate complete intensity, 3: high intensity). The subtype of these tissues are Luminal type. Expression was observed in the cytoplasm and cell membrane **(B**). Pathological response rate of LAT1 positive or negative in ER + HER2- or ER-HER2- breast cancer patients with neo adjuvant chemotherapy. P < 0.0001 for pCR rate in ER + HER2- patients, P = 0.0016 for pCR in ER-HER2-patients. Pre LAT1 total score of 14 or more was positive. The cutoffs for positive was defined as ROC curve of CMR or non-CMR **(C**). LAT1 was highly expressed in breast carcinoma cells but not in adjacent normal or non-neoplastic breast tissues**(D**)**.**The subtype of this tissue is triple negative.

Representative images of LAT1 immunohistochemistry are illustrated in Fig. 1A. Among the breast cancer patients who received NAC, 129 (90.2%) were immunohistochemically positive for LAT1 (immunohistochemical staining of LAT1was analyzed using the intensity scoring system, 0–3, and an intensity score ≥ 1 was defined as positive (Fig. 1B). The mean LAT1 score was 14.5 (range, 0–30).

The correlations between LAT1 expression in carcinoma cells and clinicopathological variables are summarized in Table 2. The LAT1 status pre-NAC (Pre) was significantly higher in patients with cT1 or cT2 disease (15.4 vs 11.6, *p* = 0.0403) and ER-negative patients (16.9 vs 13.1, *p* = 0.0229). LAT1 expression was not significantly associated with other factors. The Pre-LAT1 score in the high Ki-67 (post) group was significantly higher than that in the low Ki-67 (post) group (15.5 vs 11.6, *p* = 0.0301, Table 2).

**Table 2:** Clinicopathological factors with the status of LAT1 (n = 143).

	Pre LAT1			PostLATl		
	Total score			Total score		
	Mean	SD	*P* value	Mean	SD	*P* value
Total	14.5 (0–30)	9.71		10.6 (0–30)	7.74	
**Pre cT**						
1/2	15.4	0.91	0.0403*	11.2	0.83	0.1570
3/4	11.6	1.67		8.8	1.43	
**PrecN**						
0	18.1	2.16	0.0806	12.4	2.23	0.4091
1 <	14.0	0.86		10.4	0.76	
**Pre cStage**						
O/I/II	15.4	0.94	0.0867	11.3	0.85	0.1530
nr/vi	12.3	1.54		9.0	1.34	
**Pre ER**						
Positive	13.1	1.02	0.0229*	10.3	0.87	0.5293
Negative	16.9	1.29		11.3	1.27	
**Pre PR**						
Positive	13.2	1.11	0.0831	9.7	0.92	0.1185
Negative	16.0	1.16		12.0	1.13	
**Pre HER2**						
Positive	16.8	1.58	0.0967	9.5	0.83	0.3792
Negative	13.7	0.93		11.0	1.46	
**PostKi-67 LI**						
High	15.5	1.17	0.0301*	12.2	0.94	0.0256*
(Cut off 9.6) Low	11.6	1.34		9.0	1.07	
**Post SUV max**						
High	15.8	1.44	0.0140*	10.7	1.41	0.6198
(Cut off 2) Low	20.1	0.89		9.8	1.02	

LAT1, L-type amino acid transporter-1, SLC7A5; ER, estrogen receptor; PR, progesterone receptor; HER2, human epidermal growth factor receptor-2 ; SUV, standardized uptake value.

The cutoffs of Ki-67 LI and SUVmax was defined as ROC curve of Pre LAT1 intensity score 2 < as positive.

We assessed the correlation between LAT1 expression and SUVmax, an indicator of glucose metabolism. Post SUVmax-low patients had significantly higher Pre-LAT1 scores than Post SUVmax-high patients (Pre 20.1 vs 15.8, *p* = 0.0140, Table 2). Moreover, we compared the differences between pathologic response and CMR in the cases in which data were available (n = 94; Table 3). Even in CMR cases, 62.7% (32/51) did not achieve pCR following NAC. In addition, ER-positive/HER2-negative and Pre-LAT1-positive cases were significantly associated with not achieving pCR (*p* = 0.0150 and *p* = 0.0374, respectively; Table 3).

**Table 3:** Comparison of treatment response assessments between pathologic response and CMR(PERCIST) in breast cancer patients with neoadjuvant chemotherapy (n = 94).

	non-PCR		pCR		Total	*p* value
	n	(%)	n	(%)		
CMR	32	(62.7)	19	(37.3)	51	
**Pre subtype**						
ER + HER2-	17	(89.4)	2	(10.6)	19	0.0150*
ER + HER2 +	5	(55.5)	4	(44.5)	9	
HER2 enriched	4	(57.1)	3	(42.9)	7	
TNBC	6	(37.5)	10	(62.5)	16	
Pre LAT1 expression positive	31	(67.3)	15	(32.7)	46	0.0374*
Negative	1	(20.0)	4	(80.0)	5	
non-CMR	39	(90.6)	4	(9.4)	43	
**Pre subtype**						
ER + HER2-	23	(92.0)	2	(8.0)	25	0.8150
ER + HER2 +	5	(83.3)	1	(16.7)	6	
HER2 enriched	4	(100)	0		4	
TNBC	7	(87.5)	1	(12.5)	8	
Pre LAT1 expression positive	28	(87.5)	4	(12.5)	32	0.2182
Negative	11	(100)	0		11	
Total	71		23		94	

CMR, complete metabolic response; PERCIST, Positron Emission Tomography Response Evaluation Criteria in Solid Tumors; ER, estrogen receptor; PR, progesterone receptor; HER2, human epidermal growth factor receptor-2; TNBC, triple negative breast cancer; LATl, L-type amino acid transporter-1, SLC7A5; Pathological complete response: pCR.

We then examined whether the status of Pre-LAT1 could predict the response to NAC. We classified the HER2-negative patients (n = 106) into four groups according to Pre-LAT1 positivity (cut off TS14; positive, n = 60 and negative, n = 46) and ER positivity to compare the pCR rate (Fig. 1C). Among patients with ER-positive/HER2-negative tumors, the Pre-LAT1-positive group had a significantly lower pCR rate than the Pre-LAT1-negative group (5.56% vs 12.90%, *p* < 0.0001). In patients with ER-negative/HER2-negative tumors, the Pre-LAT1-positive group had a significantly higher pCR rate than the Pre-LAT1-negative group (45.83% vs 33.33%, *p* = 0.0016). These findings indicated that the Pre-LAT1 status was significantly correlated with response to chemotherapy in both ER-positive and HER2-negative breast cancer patients.

### LAT1 expression in breast carcinoma cells

**Figure 2:**
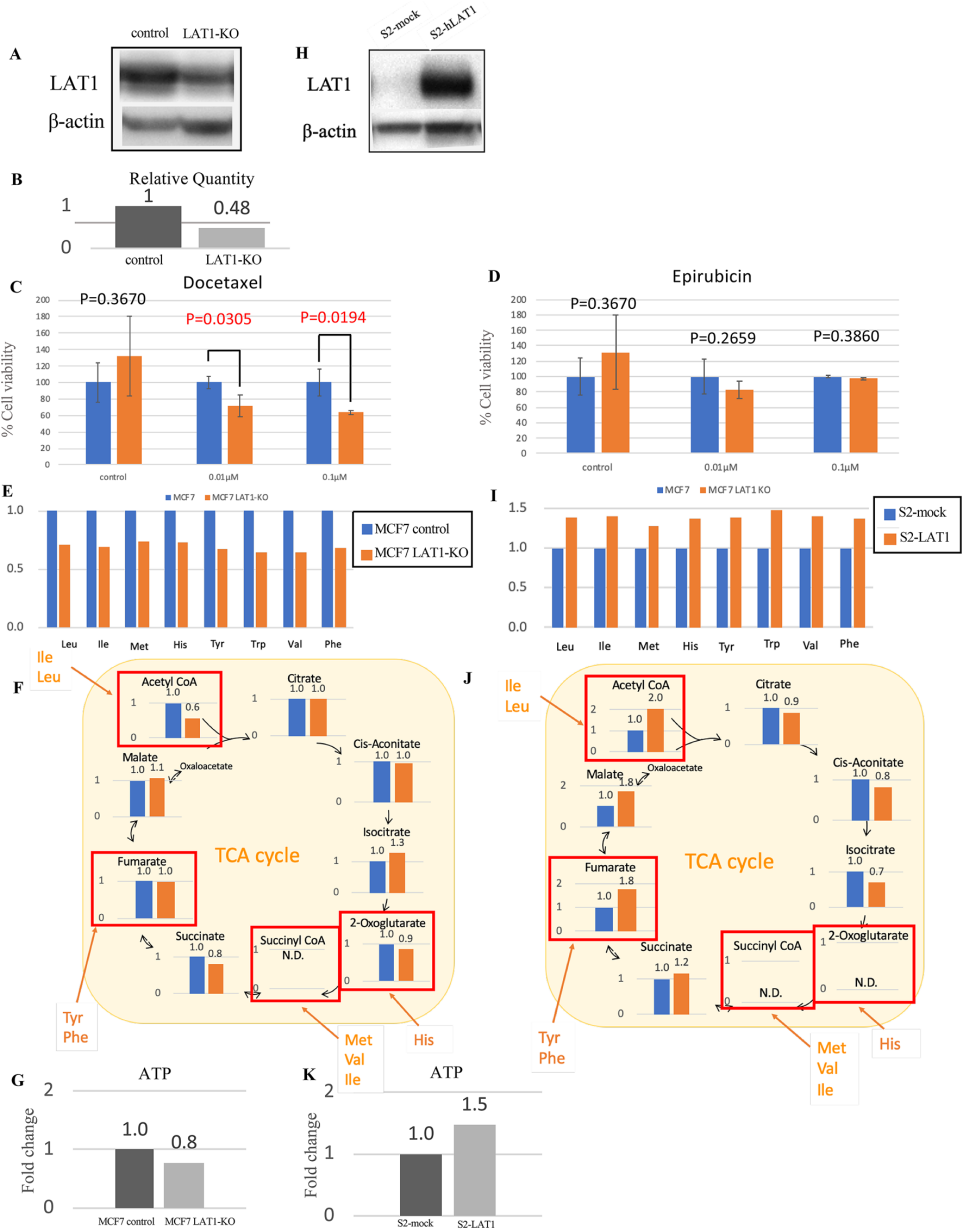
In vitro, MCF7 LAT1 knockout cell and these relative quantity (**A,B**). Cell proliferation in MCF7 control or LAT1-KO cell with 0, 0.01, 0.1 μM docetaxel (**C**) or epirubicin (**D**) for 48 h. Metabolome analysis of control and LAT1-KO cells. These are amino acid levels uptake via LAT1 with control set to 1(**E**), intermediates of TCA cycle (fold change) (**F**) and ATP levels (fold change) (**G**). Western blot data of LAT1 protein in S2-mock and S2-LAT1(**H**). Metabolome analysis of S2-mock and S2-LAT1 cells. These are amino acid levels uptake via LAT1 with control set to 1(**I**), intermediates of TCA cycle (fold change)(**J**) and ATP levels (fold change) (**K**).

As the clinical study indicated that LAT1 was involved in drug resistance in ER-positive tumors, we used MCF7 cells to examine the biological function of LAT1 following drug treatment. To assess the function of LAT1, we knocked out LAT1 using guide RNAs (Fig. 2A,B). We then performed cell proliferation experiments to evaluate the responsiveness of the LAT1-knockout MCF7 cells to chemotherapy (epirubicin and docetaxel). Cell proliferation and drug-induced cell toxicity were measured using the WST-8 assay in cells treated with drugs for 48 h. LAT1-knockout MCF-7 cells were significantly more sensitive to docetaxel than were the control cells (Fig. 2C) but this tendency was not detected when studying the sensitivity to epirubicin (Fig. 2D).

In addition, we also performed metabolomic analysis using both LAT1-knockout MCF7 cells and -overexpressing cells to explore the correlation between LAT1 status and cancer metabolism (Supplementary data). The LAT1-overexpressing cells established from MCF7 cells were unavailable and therefore we used S2-mock and S2-LAT1 cells which lost the cellular properties of tubular formation and contributed to drug discovery as normal-type and tumor-type stable cell lines. In LAT1-knockout cells, the levels of amino acids transported by LAT1 were significantly reduced, with an associated decline in supplemented TCA intermediates and ATP levels (Fig. 2E–G). Conversely, LAT1-overexpressing cells had increased levels of amino acids and an associated increase in supplemented TCA intermediates and ATP production (Fig. 2H–K).

### Changes in glucose and amino acid metabolism before and after chemotherapy

**Figure 3:**
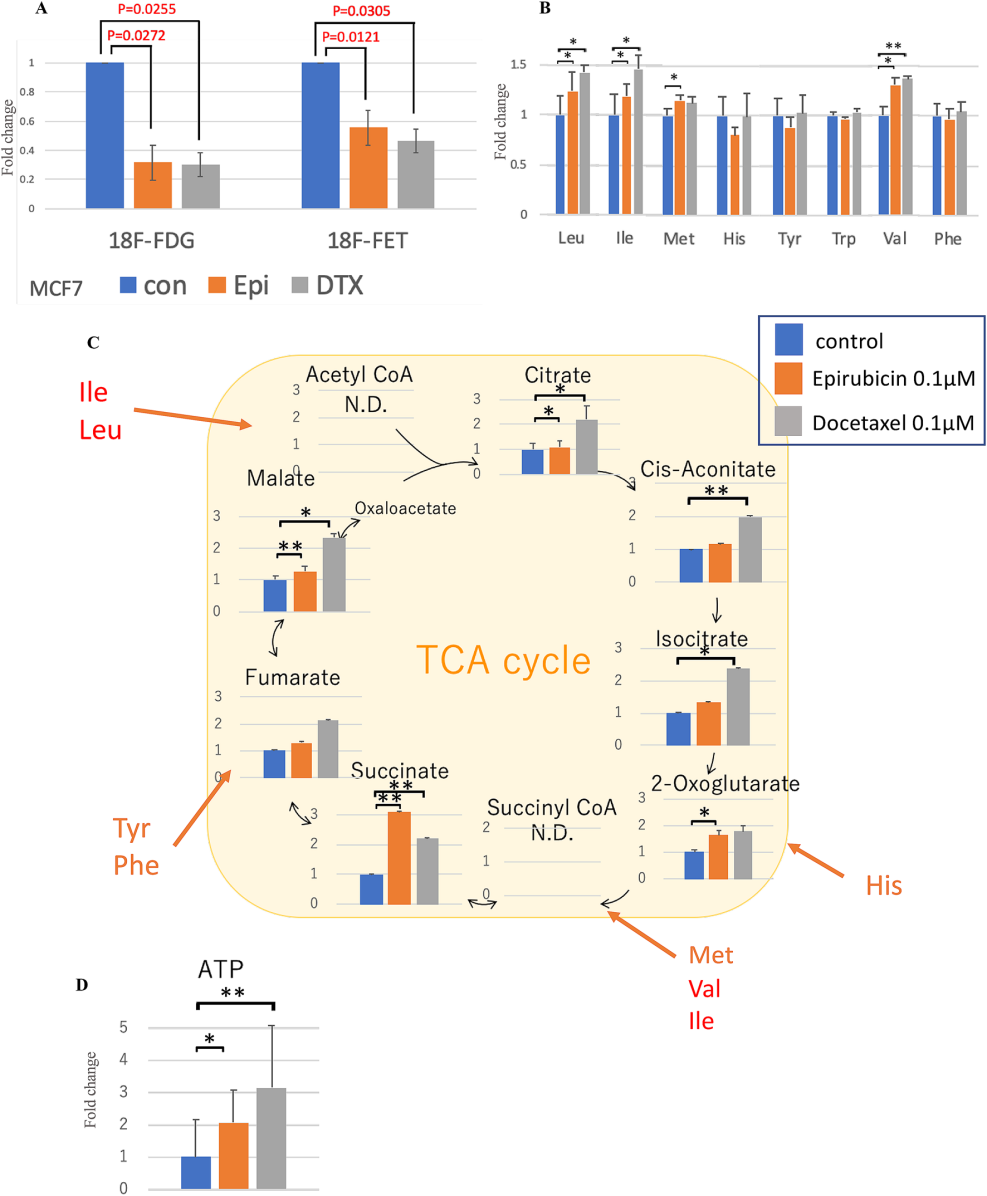
Change of amino acid metabolism with chemotherapy. Cellular uptake of radiotracers 18F-FDG and 18F-FET in MCF7 cells with 0.1 μM epirubicin or docetaxel for 48 h (**A**). Metabolome analysis of MCF7 cells with 0.1 μM epirubicin or docetaxel for 48 h. These are amino acid levels uptake via LAT1 with control set to 1 (**B**), intermediates of TCA cycle (fold change) (**C**) and ATP levels (fold change) (**D**). bars, N.D., the metabolite concentration was below the detection limit of the analysis. All the P values were evaluated by the Student t-test. *P < 0.05; **P < 0.01; ***P < 0.001.

To further explore the potential impact of glucose and amino acid metabolism in breast carcinoma cells, we evaluated 18F-FDG and 18F-FET uptake following the administration of chemotherapeutic agents. Both 18F-FDG and 18F-FET uptake were significantly reduced by epirubicin and docetaxel (FDG: *p* = 0.0272 and *p* = 0.0255, respectively; FET: *p* = 0.0121 and *p* = 0.0305, respectively; Fig. 3A). The metabolomic analysis of MCF7 cells with chemotherapy revealed that the levels of leucine, isoleucine, and valine, which are transported by LAT1 and are all branched-chain amino acids (BCAAs), were markedly increased (Supplementary data, Fig. 3B). In addition, these cells had increased TCA intermediates and ATP production (Fig. 3C,D).

## Discussion

In this study, we first evaluated the expression of LAT1, an amino acid transporter whose expression has been reported to increase in cancer^25^. We found that LAT1 could be an indicator of amino acid metabolism in breast cancer patients. As summarized in Table 2, it was expressed in carcinoma cells but not in adjacent normal or non-tumorous mammary tissue (Fig. 1D), which is consistent with the results of previous studies^21^. In addition, its status has been reported to be significantly correlated with tumor size, nuclear grade, and Ki-67 LI in breast cancer^26^. Therefore, the results of this study, as well as these previous results, indicate that LAT1 levels in breast carcinoma cells could represent the status of proliferation in breast cancer, although only after chemotherapy. Moreover, these results demonstrate that LAT1 may be a predictor of clinical prognosis in breast cancer patients as well as a predictive factor of the therapeutic efficacy of preoperative and postoperative chemotherapy. However, further investigations are required to confirm whether LAT1 could substitute for the Ki-67 LI for predicting clinical outcome and therapeutic effects among breast cancer patients with different subtypes.

In addition, we examined the association between amino acid and glucose metabolism in breast cancer. Patients with lower glucose metabolism after therapy had higher LAT1 expression before therapy, indicating that amino acid metabolism was enhanced in patients with low glucose metabolism following chemotherapy. Even in patients who lost all FDG uptake, more than half had residual tumors (Table 3). These results indicated that amino acid metabolism could allow tumors to survive despite markedly decreased glucose uptake after treatment. Further, we clarified that amino acid metabolism and glucose metabolism are closely correlated in breast cancer and showed that amino acid metabolism may be involved in chemoresistance, but further investigations are required for clarification.

ER signaling has been reported to regulate LAT1 in ER-positive breast cancer cell lines under nutritional stress^24^. Additionally, the association of abundant *LAT1* with adverse clinical outcome was reported in ER-positive patients undergoing endocrine therapy^24,27^. However, the correlation between chemotherapy and amino acid metabolism has not been reported in breast cancer. Here, we examined 106 cases of ER-positive/HER2-negative breast cancer undergoing NAC, and the pCR rate was significantly lower in patients with high Pre-LAT1. NAC is more effective in patients with ER-negative/high Ki-67 LI tumors^28,29^. We observed that the pCR rate was high in LAT1-positive patients and was correlated with Ki-67 LI in patients with ER-negative tumors. Conversely, in patients with ER-positive tumors, the pCR rate was significantly lower in LAT1-positive patients, indicating that LAT1 could be related to treatment resistance in ER-positive breast cancer. Moreover, this indicated that ER could play a pivotal role in LAT1 regulation in breast cancer, as seen with AR in prostate cancer^30^.

We used LAT1-knockout breast cancer cells to further explore the role of LAT1 in chemoresistance. The MCF7 LAT1-knockout cell line had increased sensitivity to docetaxel. These results indicate the possibility of combining LAT1 inhibitors and chemotherapy in ER-positive/HER2-negative breast cancer patients. When epirubicin was administered at 0.01 µM, there were no significant differences in sensitivity, although the cell proliferation tended to decrease and at an epirubicin dose of 0.1 µM, no significant differences were either detected because only a few cells survived in both control and drug administration.

We performed a metabolomic analysis of LAT1-knockout and -overexpressing cells to further clarify the changes in amino acid metabolism and glucose metabolism based on LAT1 expression. The amino acid levels before and after chemotherapy have been compared in breast cancer^24,27^; however, to the best of our knowledge, the correlation with the TCA cycle has not been studied. We found that LAT1-knockout cells had lower levels of amino acids transported via LAT1 and decreased levels of TCA cycle intermediates, leading to reduced ATP production. Conversely, overexpression of LAT1 increased the levels of intracellular amino acid transported via LAT1 and increased TCA cycle intermediates, which enhanced ATP production. These results indicated that LAT1 promotes amino acid uptake, which contributed to energy production by supplying amino acids into the TCA cycle. However, further investigations are required for clarification.

Of particular interest was the fact that the changes in cancer metabolism associated with chemotherapy were different from those observed with LAT1 knockout or overexpression. The levels of amino acid transported via LAT1, leucine, isoleucine, and valine, were significantly increased. These amino acids are all BCAAs, which play an important role in protein synthesis and are an energy source in cancer^31^. In addition, tumor cells preferentially uptake BCAAs ^31,32^, although the mechanisms driving this preference are unknown. Our metabolomic analysis revealed that epirubicin and docetaxel significantly increased succinate. Succinyl-CoA, which is derived from valine and isoleucine, is readily converted to succinate and ATP^33^. Therefore, a remarkable increase in succinate under treatment is pivotal in ATP production. Our results indicated that TCA intermediates and their source BCAAs play an important role in energy production and drug resistance after chemotherapy. A previous study demonstrated that leucine imported by LAT1 was involved in tamoxifen resistance in ER-positive breast cancer^24^. Our results suggest that all BCAAs were involved in chemoresistance and that cells may develop endocrine resistance and chemoresistance via different mechanisms. In addition, decreased FDG uptake is associated with reduced glucose metabolism^34^, but FET is a tyrosine derivative and therefore does not necessarily reflect the enhancement of amino acid metabolism mediated by LAT1. Further investigations including the use of the tracers targeting BCAAs are required to clarify the clinical significance of this finding.

There are several limitations in this study. First, a significant correlation was detected between ER and LAT1 but we only studied ER-positive cells in this study. Therefore, it awaits further investigations employing ER-negative cells and other cells derived from different subtypes as control in cell proliferation assay and cellular uptake of radiotracers in order to further substantiate the findings. In addition, the results should be also validated by long term cell survival and proliferation assays such as EdU/BrdU assay. Secondly, we should establish LAT1-overexpressed MCF7 cells and further studies are definitively warranted to validate the results of our present study.

## Conclusion

In summary, we first revealed the correlation between amino acid and glucose metabolism in breast cancer patients. The results indicated that LAT1 was involved in drug resistance, and its potential inhibition could provide a novel approach to overcome chemoresistance in ER-positive/HER2-negative breast cancer.

## Methods

### Breast cancer patients—cohort and sample selection

We retrieved clinical data from 143 breast cancer patients who received NAC and surgery at the Tohoku University Hospital and Tohoku Kosai Hospital, both in Sendai, Japan, between 2012 and 2017 and used biopsy specimens prior to chemotherapy and surgical specimens. In this study, pCR was defined as the absence of all invasive carcinoma cells and lymph node metastasis, regardless of the presence or absence of noninvasive cancer cells^35^.

### Immunohistochemistry

We performed immunohistochemistry on 5-µm-thick 10% formalin-fixed paraffin-embedded tissues. The slides were placed in an autoclave (Tomy SX-500 High-Pressure Steam Sterilizer, Tomy Seiko Co., Ltd., Tokyo, Japan) in citrate buffer (Agilent technologies, Santa Clara, USA) at 121 °C for 5 min. Tissue sections were subsequently incubated for 30 min at 25 °C in a blocking solution of 10% rabbit serum (Nichirei Biosciences, Tokyo, Japan) and incubated overnight at 4 °C with a LAT1 mouse monoclonal antibody (1:200, KE023 Trans Genics, Hyogo, Japan). The slides were incubated with a biotinylated secondary rabbit anti-mouse antibody (Nichirei Bioscience, Tokyo, Japan) at a dilution of 1:100 for 30 min at 25 °C, and peroxidase-conjugated avidin (Nichirei Bioscience, Tokyo, Japan) was used. Human placenta and skin tissues were used as positive and negative controls, respectively.

Immunohistochemical staining was evaluated using an intensity score (0: no membranous staining, 1: low intensity, 2: weak to moderate intensity, 3: high intensity) multiplied by the positive ratio per tumor area from 0 to 10, resulting in a score from 0 to 30^36,37^.

### FDG-PET/CT image acquisition protocol

Ninety-four patients with breast cancer underwent FDG-PET/CT imaging before and after chemotherapy prior to surgical resection. PET/CT was performed after at least 5 h of fasting, and FDG (3.8 MBq/kg) was administered intravenously. Then, a whole-body PET/CT scan (Biograph16, Siemens, Erlangen, Germany) was acquired 75 min after FDG injection. Two radiologists/nuclear medicine physicians independently evaluated the PET/CT images.

### Cell lines and culture

The human breast carcinoma cell lines MCF-7 cells obtained from the American Type Culture Collection (ATCC) were maintained in RPMI-1640 medium (Sigma-Aldrich, St Louis, MO, USA) supplemented with 10% fetal bovine serum (Nichirei Biosciences, Tokyo, Japan) and 100 μg/mL penicillin/streptomycin (Invitrogen, Carlsbad, CA, USA). Cells were incubated at 37 °C in 5% CO_2_.

### Western blotting

Western blotting was performed as reported previously^38^. Primary antibodies against LAT1 (1:250, Cell Signaling Technology Japan, K.K., Tokyo, Japan) and β-actin (1:10,000, Proteintech, Tokyo, Japan) were used.

### Knockout of LAT1 in breast carcinoma cells

Genome editing was performed as previously described^39,40^using sgRNAs (CAGCCTGGCGGGCTATTCCC and TTTACGTTCCTGGACCATCC) designed to generate DNA double-strand breaks in exon 1 of human *LAT1*. sgRNAs were integrated into a publicly available gRNA cloning vector. The deletion of the exon 1 sequences was confirmed by polymerase chain reaction (Forward primer: GTACCCCAAACTCCATCCTTGG, Reverse primer: ATGAAAGAAACGCACGTGGTTT). Only heterozygous knockout cell lines were obtained.

### Overexpression of LAT1

We used cells stably expressing hLAT1 (S2-LAT1), a mouse cell line transfected with the vector pcDNA 3.1-hLAT1, in this study. Control cells (S2-mock) were transfected with the empty pcDNA3.1 vector. Details were previously reported^41^. These cell lines have lost the tubular properties and contributed to drug discovery as tumor-type and normal-type stable cell lines.

### WST-8 assay: cell proliferation assay

Breast carcinoma cells were cultured in 96-well plates for 24 h, at which point we added epirubicin (Wako, Tokyo, Japan), docetaxel (FUJIFILM Wako Pure Chemical Corporation, Osaka, Japan), or dimethyl sulfoxide (DMSO) (FUJIFILM Wako Pure Chemical Corporation, Osaka, Japan) as a control. After 48 h, the cell number was measured as previously reported^42^.

### Metabolomic analysis by capillary electrophoresis time-of-flight mass spectrometry

Cells in a 100-mm dish were washed by 5% mannitol, and then 1 mL methanol(Wako, Tokyo, Japan) with 25 μM each l-methionine sulfone (Wako, Tokyo, Japan), 2-morpholinoethanesulfonate (Dojindo, Kumamoto, Japan), and D-Camphor-10-sulfonic Acid Sodium Salt (Wako, Tokyo, Japan) was added. Collected cell and solution with scraper, and the transferred solution was mixed with Milli-Q water and chloroform. The solution was centrifuged, and the separated aqueous layer was centrifugally filtered through an HMT 5-kDa ultrafiltration tube (Human Metabolome Technologies Inc., Japan). Samples were dried using an evacuated centrifuge, and Milli-Q water containing 3-aminopyrrolidine (Sigma Aldrich, USA) and Trimesate (Wako, Tokyo, Japan) was added. Metabolomic analyses were performed by capillary electrophoresis time-of-flight mass spectrometry at the Institute for Advanced Biosciences, Keio University (Tsuruoka, Japan) as previously reported^43^. The measured metabolite concentrations were normalized using cell number to obtain the amount per cell. Raw data were processed using software (MasterHands) developed in-house as previously described^43^.

### Cellular uptake of radiotracers

Twenty-four-well plates were seeded with 5.0 × 10^5^ cells per well. After incubation for 24 h, 100 nM epirubicin, docetaxel, or an equivalent volume of DMSO was added to each well and incubated for 48 h. The medium was subsequently changed to glucose-free medium (DMEM, no glucose, 0.5% BSA, 0.125 mM sodium ascorbate) with 50 µCi/mL FDG or FET and incubated for 1 h. The cells were washed three times with PBS, lysed, and suspended in 0.5 M aqueous sodium hydroxide and 0.5 M hydrochloric acid. Radioactivity was measured with a γ‐counter (AccuFLEX γ7000; Hitachi Aloka Medical, Tokyo, Japan). Total protein concentration in the samples was determined using a protein assay (Qubit: Thermo Fisher Scientific, Tokyo, Japan).

### Statistical analyses

Statistical analysis was carried out using JMP Pro 14 software (SAS Institute, Inc., Cary, NC, USA). Pearson’s chi-square test or Fisher’s exact test was performed for clinicopathological and cell viability analyses. We used receiver operating characteristic (ROC) curve analysis to calculate the optimal cutoff values of Ki-67 LI and SUVmax. The pathological response rate was analyzed using the McNemar test, and the cutoffs for LAT1 positivity were defined using a ROC curve of CMR. P values of metabolome analysis were calculated using the two-tailed Student’s t-test. All p values of less than 0.05 were considered statistically significant in this study.

### Ethical approval

All procedures involving human participants were in accordance with the ethical standards of the institutional and/or national research committee and with the 1964 Helsinki Declaration and its later amendments or comparable ethical standards.

### Informed consent

The retrospective study was approved by the institutional review board of Tohoku University Hospital (No. 2015-1-520) and Kosai Hospital with a waiver for the need to obtain informed consent.

## Supplementary Information

Supplementary Dataset.

Supplementary Figures.

## Data Availability

Authors can confirm that all relevant data are included in the article and/or its supplementary information files.
