# Supplementary material for: L-type amino acid transporter 1 is associated with chemoresistance in breast cancer via the promotion of amino acid metabolism
Source: Sci Rep. 2021 Jan 12;11:589. doi: 10.1038/s41598-020-80668-5 (PMC7803739; doi:10.1038/s41598-020-80668-5)
Supplement: Supplementary file 2 — Supplementary Figures. [file 41598_2020_80668_MOESM2_ESM.pdf]

# L-type amino acid transporter 1 is associated with chemoresistance in breast cancer via the promotion of amino acid metabolism

Miku Sato[1,2], *Narumi Harada-Shoji[1], Takafumi Toyohara[3], Tomoyoshi Soga[4], Masatoshi Itoh[5], Minoru Miyashita[1], Hiroshi Tada[1], Masakazu Amari[6], Naohiko Anzai[7], Shozo Furumoto[8], Takaaki Abe[3], Takashi Suzuki[9], Takanori Ishida[1], Hironobu Sasano[2]

[1]Department of Breast and Endocrine Surgical Oncology, Tohoku University Graduate School of Medicine, 1-1 Seiryo-machi, Aoba-ku, Sendai 980-8574, Japan.

[2]Department of Pathology, Tohoku University Hospital, 1-1 Seiryo-machi, Aoba-ku, Sendai 980-8574, Japan.

[3] Department of Applied Physics, Graduate School of Engineering, Tohoku University, 1-1 Seiryo-machi, Aoba-ku, Sendai 980-8574, Japan.

[4] Institute for Advanced Biosciences, Keio University, Tsuruoka, Yamagata, 997-0035, Japan

[5] Sendai Medical Imaging Center, 2-1-25 Itsutsubashi, Aoba-ku, Sendai 980-0022, Japan.

[6] Department of Breast Surgery, Tohoku Kosai Hospital, 2-3-11 Kokubuncho, Aoba-ku, Sendai 980-0803, Japan.

[7] Department of Pharmacology, Chiba University Graduate School of Medicine, 1-8-1 Inohana, Chiba 260-0856, Japan

[8] Cyclotron and Radioisotope Center, Tohoku University School of Medicine, 6-3 Aramaki-aza-Aoba, Aoba-ku, 980-8578 Sendai, Japan.

[9] Department of Pathology and Histotechonology, Tohoku University Graduate School of Medicine, Tohoku University Hospital, 1-1 Seiryo-machi, Aoba-ku, Sendai 980-8574, Japan.

Fig2A (LAT1)

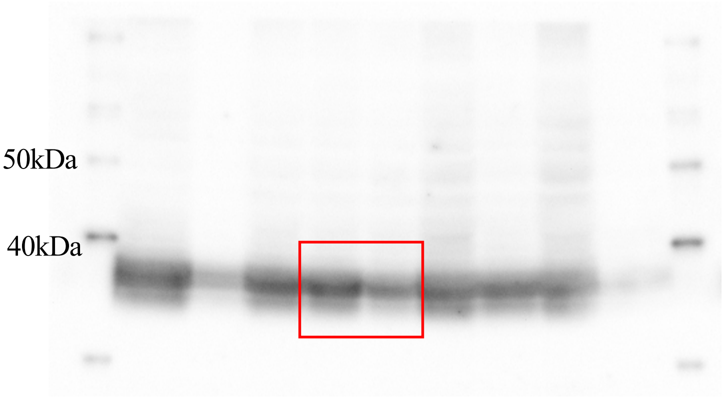


Fig 2A (β-actin)

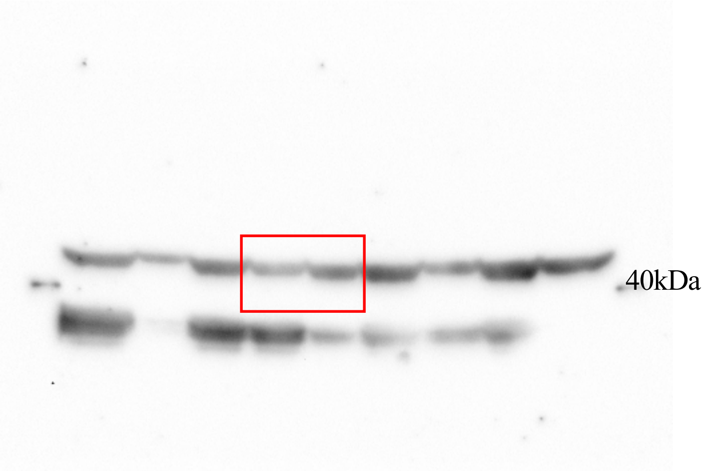


Fig 2H (LAT1)

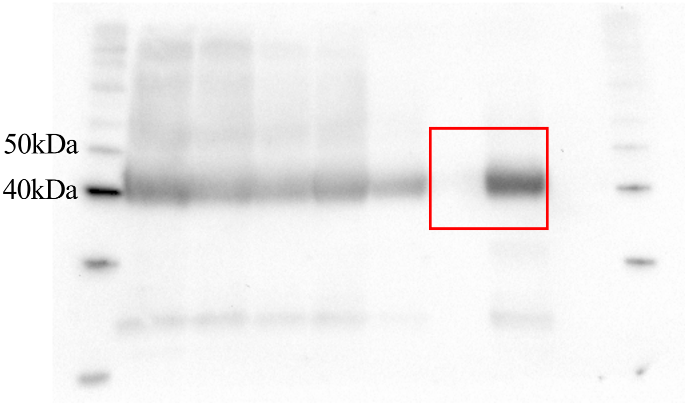


Fig 2H (β-actin)

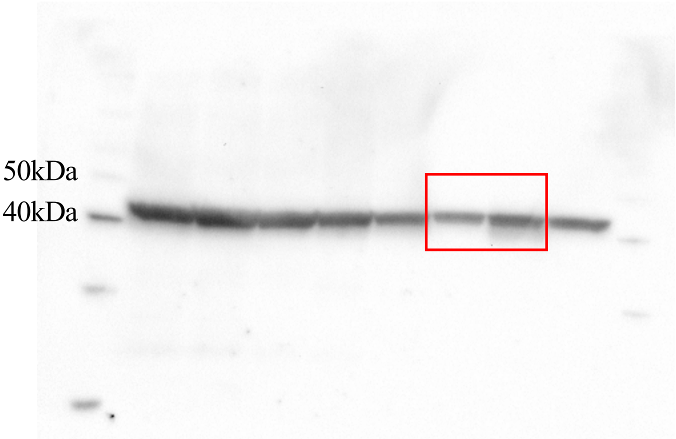


* β-actin is the same membrane of LAT1 that was washed and reprobed.
